# The Effect of Aortic Angulation on Clinical Outcomes of Patients Undergoing Transcatheter Aortic Valve Replacement

**DOI:** 10.21470/1678-9741-2022-0436

**Published:** 2024-02-21

**Authors:** Adem Aktan, Muhammed Demir, Tuncay Güzel, Mehmet Zülküf Karahan, Burhan Aslan, Raif Kılıç, Serhat Günlü, Bayram Arslan, Mehmet Özbek, Faruk Ertaş

**Affiliations:** 1 Department of Cardiology, Mardin Training and Research Hospital, Mardin, Turkey; 2 Department of Cardiology, Faculty of Medicine, Dicle University, Diyarbakır, Turkey; 3 Department of Cardiology, Health Science University, Gazi Yaşargil Training and Research Hospital, Diyarbakır, Turkey; 4 Department of Cardiology, Faculty of Medicine, Artuklu University, Mardin, Turkey; 5 Department of Cardiology, Diyarlife Hospital, Diyarbakır, Turkey; 6 Department of Cardiology, Dağkapı State Hospital, Diyarbakır, Turkey; 7 Department of Cardiology, Ergani State Hospital, Diyarbakır, Turkey

**Keywords:** Aortic Angulation, Aortic Stenosis, Transcatheter Aortic Valve Replacement, Logistic Models

## Abstract

**Introduction:**

The aim of this study was to assess the impact of aortic angulation (AA) on periprocedural and in-hospital complications as well as mortality of patients undergoing Evolut™ R valve implantation.

**Methods:**

A retrospective study was conducted on 264 patients who underwent transfemoral-approach transcatheter aortic valve replacement with self-expandable valve at our hospital between August 2015 and August 2022. These patients underwent multislice computer tomography scans to evaluate AA. Transcatheter aortic valve replacement endpoints, device success, and clinical events were assessed according to the definitions provided by the Valve Academic Research Consortium-3. Cumulative events included paravalvular leak, permanent pacemaker implantation, new-onset stroke, and in-hospital mortality. Patients were divided into two groups, AA ≤ 48° and AA > 48°, based on the mean AA measurement (48.3±8.8) on multislice computer tomography.

**Results:**

Multivariable logistic regression analysis was performed to identify predictors of cumulative events, utilizing variables with a P-value < 0.2 obtained from univariable logistic regression analysis, including AA, age, hypertension, chronic renal failure, and heart failure. AA (odds ratio [OR]: 1.73, 95% confidence interval [CI]: 0.89-3.38, P=0.104), age (OR: 1.04, 95% CI: 0.99-1.10, P=0.099), hypertension (OR: 1.66, 95% CI: 0.82-3.33, P=0.155), chronic renal failure (OR: 1.82, 95% CI: 0.92-3.61, P=0.084), and heart failure (OR: 0.57, 95% CI: 0.27-1.21, P=0.145) were not found to be significantly associated with cumulative events in the multivariable logistic regression analysis.

**Conclusion:**

This study demonstrated that increased AA does not have a significant impact on intraprocedural and periprocedural complications of patients with new generation self-expandable valves implanted.

**Table:** 

Abbreviations, Acronyms & Symbols				
AA	= Aortic angulation		LVESD	= Left ventricular end-systolic diameter
AR	= Aortic regurgitation		MI	= Myocardial infarction
AS	= Aortic stenosis		MR	= Mitral regurgitation
AV	= Aortic valve		MSCT	= Multislice computed tomography
AVA	= Aortic valve area		NCC	= Non-coronary cusp
BE	= Balloon-expandable		NYHA	= New York Heart Association
CABG	= Coronary artery bypass grafting		OR	= Odds ratio
CAD	= Coronary artery disease		PCI	= Percutaneous coronary intervention
CAU	= Caudal		PPMI	= Permanent pacemaker implantation
CI	= Confidence interval		PVL	= Paravalvular leak
COPD	= Chronic obstructive pulmonary disease		RAO	= Right anterior oblique
CRA	= Cranial		RCA	= Right coronary artery
CVE	= Cerebrovascular event		RCC	= Right coronary cusp
IQR	= Interquartile range		SE	= Self-expandable
IVSDD	= Interventricular septum diastolic diameter		SPAP	= Systolic pulmonary artery pressure
LAD	= Left atrial diameter		STS	= Society of Thoracic Surgeons
LAO	= Left anterior oblique		TAVR	= Transcatheter aortic valve replacement
LBBB	= Left bundle branch block		TR	= Tricuspid regurgitation
LVEDD	= Left ventricular end-diastolic diameter		VARC-3	= Valve Academic Research Consortium-3
LVEF	= Left ventricular ejection fraction			

## INTRODUCTION

Transcatheter aortic valve replacement (TAVR) has emerged as an alternative treatment to surgery in inoperable or high-risk patients with severe aortic valve disease^[1]^. Nowadays, transcatheter treatment is an alternative to surgical valve replacement not only in high-risk patients, but also in those with lower or intermediate risk (especially over 70°)^[2]^. With advancements in implantation techniques and prosthetic valves, TAVR complications have significantly decreased in recent years^[3]^.

TAVR with self-expandable (SE) valves has been shown to be effective in treating severe aortic stenosis (AS) with fewer long-term complications, including relatively less annular rupture, vascular complications, and paravalvular leak (PVL)^[4]^. However, the presence of a horizontally oriented aortic root during TAVR with the SE Evolut™ R valve may pose challenges due to its rigidity and lack of orientability^[4]^. Therefore, a thorough analysis of the aortic valvular complex and multislice computed tomography (MSCT) before the procedure is crucial for patients undergoing SE Evolut™ R TAVR^[5]^. This allows for accurate measurement of aortic valve calcification, precise reconstruction of the aortic annulus, determination of the aortic angulation (AA), and appropriate selection of the bioprosthesis^[6]^. AA refers to the measurement of the angle between the horizontal plane and the plane of the aortic annulus. It is a term used to describe the degree of deviation or tilt of the aortic root from the horizontal position. AA can significantly impact the positioning and optimal placement of SE valves, especially when dealing with a high-angle aortic root (*e.g.*, AA > 70°)^[7]^. The presence of a high AA can pose challenges in achieving successful positioning and optimal placement of SE valves in the aortic region^[7]^. Previous studies have indicated that the presence of horizontal aortic root anatomy presents numerous challenges during the coaxial implantation of the SE valve^[7]^. These challenges may include prolonged fluoroscopy time, valve migration, aortic injury, the potential need for a second valve, left ventricular perforation, postdilatation, and the occurrence of postprocedural PVL^[7,8]^. However, a recent study found that AA grade did not significantly affect early clinical outcomes in patients who underwent TAVR with SE Evolut™ R valves^[9]^. While these studies were being conducted, patients with an extremely horizontal aorta were generally excluded from clinical trials^[5,9]^. However, a series of seven cases applying SE Evolut™ R TAVR in patients with an extremely horizontal aorta observed a high device success rate and minimal/mild PVL, along with no mortality during the three-month follow-up, using techniques based on MSCT evaluation and patient anatomy^[4]^.

There are limited studies in the literature concerning the application of TAVR with SE Evolut™ valves. The aim of this study was to assess the impact of AA on periprocedural and in-hospital complications and mortality in Evolut™ R valve implantation.

## METHODS

### Study Design

**Fig. 1:**
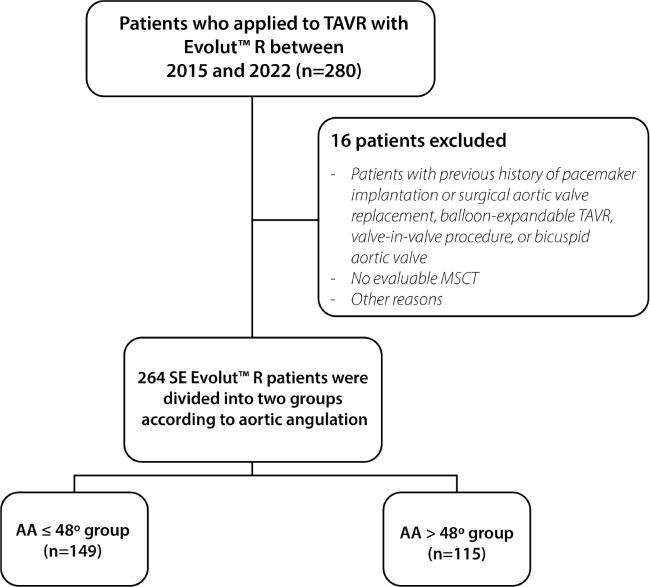
Flowchart of study population. AA=aortic angulation; MSCT=multislice computed tomography; SE=self-expandable; TAVR=transcatheter aortic valve replacement.

**Fig. 2:**
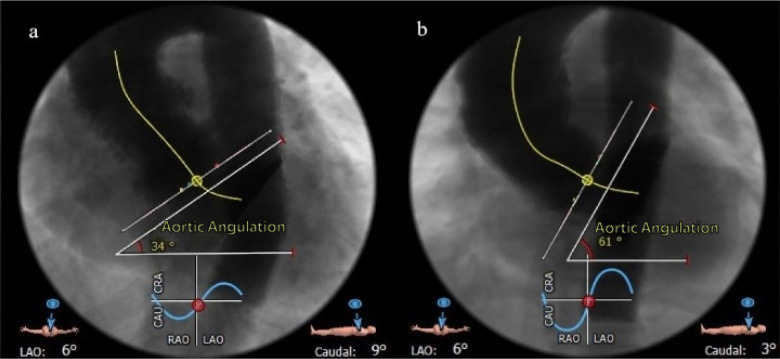
Aortic angulations (AAs) obtained in multislice computer tomography. a) AA ≤ 48°; b) AA > 48°. CAU=caudal; CRA=cranial; LAO=left anterior oblique; RAO=right anterior oblique.

This retrospective observational study aimed to review the medical records of 280 patients who underwent TAVR with transfemoral approach at our hospital from August 2015 to August 2022. After excluding 16 patients who did not meet the study criteria, a total of 264 patients were included in the analysis (Figure 1). Patients with a history of pacemaker implantation or surgical aortic valve replacement, balloon-expandable (BE) TAVR, valve-in-valve procedure, bicuspid aortic valve, no evaluable MSCT prior to TAVR, no transfemoral access, and valve-in-valve TAVR were excluded from the study. The severity of aortic valve disease was independently evaluated by at least three cardiologists, and the assessment of AS severity was conducted by a multidisciplinary cardiac team following current guidelines. The study included patients who received new generation SE Evolut™ R valves (Medtronic, Minneapolis, Minnesota, United States of America). All patients considered for inclusion underwent MSCT angiography with a minimum of 64 sections. Evaluation of AA from the coronal projection was performed using MSCT (Figure 2). Consistent with previous studies^[10]^, the study population was divided into two groups based on the mean AA to investigate the impact of AA on clinical outcomes of TAVR patients. The mean AA was 48.3±8.8, and the patients were divided into two groups: AA ≤ 48° group and AA > 48° group. Baseline demographic and clinical information, echocardiography and coronary computed tomography angiography data, procedural details, and 30-day results were collected from hospital records and compared between the two groups. TAVR endpoints, device success, and clinical events were evaluated according to the definitions recommended by the Valve Academic Research Consortium-3 (VARC-3)^[11]^. Analysis was conducted for all-cause mortality, stroke, myocardial infarction (MI), the need for permanent pacemaker, and rehospitalization. All patients provided informed consent before inclusion in the study. The study protocol was approved by the institutional review board and adhered to the principles of the Declaration of Helsinki. The study protocol was approved by the local ethics committee (Gazi Yasargil Training and Research Hospital Ethics Committee; date and number: 21/10/2022 - 214).

### Echocardiography and Electrocardiogram Analysis

Before the TAVR procedure, a standard protocol was followed using a GE Vivid 5 device (GE Medical Systems, Milwaukee, United States of America), following current European and American guidelines^[12]^. The severity of AS was assessed based on aortic valve area (AVA) calculations using peak velocity, mean gradient, and the continuity equation, as recommended by the European Society of Echocardiography guidelines. Severe stenosis was defined as AVA < 1 cm^2^ and indexed AVA (AVA/body surface area) < 0.6 cm^2^/m^2^^[7]^. Standard 12-lead electrocardiogram recordings were obtained for each patient in the supine position before and after TAVR procedure using electrocardiogram (Schiller, Bavaria, Germany) with a paper speed of 25 mm/sec and an amplitude of 10 mm/mV.

### Multislice Computed Tomography and Transcatheter Aortic Valve Replacement

All patients underwent MSCT prior to the procedure. MSCT evaluation included assessment of aortic anatomy, ascending aorta diameter, aortic annulus diameter, coronary artery locations, aortic valve structure, AVA, and the right, left, and non-coronary cusps. Additionally, AA was determined using MSCT, with AA defined as the angle between the horizontal plane and the plane of the aortic annulus (Figure 2). TAVR procedures were performed by two experienced interventional cardiologists. Postoperative device success and complications were assessed based on the VARC-3 definition^[11]^. All procedures were conducted under conscious sedation in combination with local anesthesia, and transfemoral access was utilized for all patients. Anticoagulation was achieved with unfractionated heparin (50-70 IU/kg body weight) prior to the procedure. A temporary pacemaker was placed in the right ventricle before the bioprosthetic valve implantation, and rapid pacing was employed during the implantation process. Predilatation and postdilatation decisions were determined by the clinical evaluation of the cardiovascular team, taking into consideration patient’s characteristics, structural features of the aortic valve, and angiography or imaging studies. Many variables, including the degree of stenosis in the aortic valve, valve anatomy, level of calcification, width of the aortic root, and other factors, played a role in determining the necessity of predilatation or postdilatation.

### Clinical Outcomes and Complications

Postprocedural complications, including permanent pacemaker implantation (PPMI), new-onset stroke, pericardial tamponade, arrhythmia development, acute renal failure, major bleeding, major vascular complications, procedural coronary obstruction, new-onset left bundle branch block (LBBB), PVL, peri-procedural MI, rehospitalization, and in-hospital mortality, were determined. Cumulative events, including PPMI, new-onset stroke, moderate-severe PVL, and in-hospital mortality, were also assessed.

### Follow-up and Data Collection

Follow-up data were obtained through face-to-face visits, telephone calls, and the national data recording system. The follow-up period was defined as the time from admission to our clinic for TAVR until death from any cause or the last visit to clinic.

### Statistical Analysis

Statistical analyses were performed with IBM Corp. Released 2016, IBM SPSS Statistics for Windows, version 24.0, Armonk, NY: IBM Corp. The normality of the data distribution was tested visually (with histograms and probability curves) or statistically (with Kolmogorov-Smirnov and Shapiro-Wilk tests). Continuous variables were summarized using the mean ± standard deviation or median (interquartile range) and compared using the Student’s *t*-test or Mann-Whitney U test, where appropriate. Categorical and binary variables were presented as frequency and percentage and compared using the Pearson’s Chi-square test or Fisher’s exact test, as appropriate. Univariate and multivariate logistic regression analyses were performed to identify determinants of cumulative events. Variables with a *P*-value of < 0.2 in univariate analysis were added to multivariate analysis. *P*<0.05 was considered statistically significant in the analyses.

## RESULTS

### Key Features

A total of 264 patients were included in the study, with 149 patients in the AA ≤ 48° group and 115 patients in the AA > 48° group. The mean age of the patients was 78.9±6.4 years, and the female sex accounted for 54.9% of the total. Age and sex distribution were similar between the two groups (*P*=0.726 and *P*=0.198, respectively). The mean Society of Thoracic Surgeons score was 8.6±2.8, which did not differ significantly between the groups (*P*=0.466). The majority of patients were in New York Heart Association (NYHA) class 3 (60.2%) or NYHA class 4 (36.7%), and there was no significant difference between the groups (*P*=0.498). Hypertension was the most common comorbid condition (57.6%, *P*=0.119), followed by coronary artery disease (36.7%, *P*=0.335), heart failure (34.1%, *P*=0.752), chronic kidney failure (29.2%, *P*=0.674), diabetes mellitus (24.6%, *P*=0.705), and dyslipidemia (24.2%, *P*=0.366). The prevalence of these comorbidities was similar between the two groups, with no statistically significant difference observed. Additional demographic and clinical characteristics of the patients are summarized in Table 1.

**Table 1:** Patients’ baseline demographic and clinical characteristics.

Characteristics	Aortic angulation			
	Overall	≤ 48°	> 48°	*P*-value
	n=264	n=149	n=115	
Age, years	78.9 ± 6.4	79.0 ± 6.5	78.8 ± 6.3	0.726
Sex, female, n%	145 (54.9)	87 (58.4)	58 (50.4)	0.198
Body mass index, kg/m^2^	22.1 ± 1.8	22.0 ± 1.7	22.3 ± 1.9	0.114
NYHA classification				
Class 2	8 (3.0)	3 (2.0)	5 (4.3)	0.498
Class 3	159 (60.2)	89 (59.7)	70 (60.9)	
Class 4	97 (36.7)	57 (38.3)	40 (34.8)	
STS risk score, %	8.6 ± 2.8	8.7 ± 2.7	8.5 ± 2.8	0.466
Hypertension, n%	152 (57.6)	92 (61.7)	60 (52.2)	0.119
Diabetes mellitus, n%	65 (24.6)	38 (25.5)	27 (23.5)	0.705
Dyslipidemia, n%	64 (24.2)	33 (22.1)	31 (27.0)	0.366
Coronary artery disease, n%	97 (36.7)	51 (34.2)	46 (40.0)	0.335
Previous PCI, n%	85 (32.2)	44 (29.5)	41 (35.7)	0.291
Previous CABG, n%	30 (11.4)	16 (10.7)	14 (12.2)	0.716
Prosthesis valve, n%	4 (1.5)	2 (1.3)	2 (1.7)	0.794
Peripheral artery disease, n%	7 (2.7)	3 (2.0)	4 (3.5)	0.473^*^
COPD, n%	29 (11.0)	17 (11.4)	12 (10.4)	0.802
Atrial fibrillation, n%	59 (22.3)	37 (24.8)	22 (19.1)	0.270
Previous CVE, n%	4 (1.4)	3 (2.0)	1 (0.9)	0.451
Chronic renal failure, n%	77 (29.2)	32 (27.8)	45 (30.2)	0.674
Heart failure, n%	90 (34.1)	52 (34.9)	38 (33.0)	0.752
Anemia, n%	141 (53.4)	77 (51.7)	64 (55.7)	0.521
Smoking, n%	69 (26.1)	36 (24.2)	33 (28.7)	0.406
Implanted valve size, mm	28.9 ± 3.4	29.1 ± 3.3	28.6 ± 3.6	0.203
Balloon predilatation, n%	66 (25.1)	38 (25.5)	28 (24.6)	0.861
Balloon postdilatation, n%	59 (22.4)	35 (23.5)	24 (21.1)	0.639

Data are expressed as mean ± standard deviation or frequencies (percentages) as appropriate

CABG=coronary artery bypass grafting; COPD=chronic obstructive pulmonary disease; CVE=cerebrovascular event; NYHA=New York Heart Association; PCI=percutaneous coronary intervention; STS=Society of Thoracic Surgeons

^*^Fisher’s exact test

Echocardiography and MSCT measurements of the patients are similar and are summarized in Table 2.

**Table 2:** Baseline echocardiographic and multislice computed tomography parameters.

Echocardiographic parameters	Aortic angulation			
	Overall	≤ 48°	> 48°	*P*-value
	n=264	n=149	n=115	
AV Doppler mean gradient, mmHg	48.9 ± 10.2	48.2 ± 8.8	50.0 ± 11.8	0.139
AV Doppler max. gradient, mmHg	79.7 ± 15.9	78.3 ± 14.2	81.4 ± 17.8	0.113
AV opening area (cm^2^)	0.67 ± 0.18	0.68 ± 0.17	0.66 ± 0.18	0.497
LVEF, (%)	50.8 ± 11.7	50.1 ± 11.9	51.7 ± 11.3	0.292
LVEDD, mm	4.9 (4.5-5.2)	4.9 (4.5-5.25)	4.8 (4.5-5.2)	0.560
LVESD, mm	3.6 ± 0.8	3.7 ± 0.9	3.4 ± 0.6	0.124
LAD, mm	4.5 ± 0.6	4.5 ± 0.6	4.4 ± 0.5	0.379
IVSDD, mm	1.4 ± 0.17	1.4 ± 0.18	1.4 ± 0.16	0.329
Ascending aorta diameter, mm	3.70.5	3.6 ± 0.5	3.7 ± 0.5	0.386
Moderate-severe MR, n%	79 (30.4)	47 (31.8)	32 (28.6)	0.580
Moderate-severe AR, n%	31 (12.1)	18 (12.2)	13 (11.8)	0.917
Moderate-severe TR, n%	59 (22.6)	40 (26.8)	19 (17)	0.059
SPAP, mmHg	41 (30-50)	45 (30-50)	40 (30-45)	0.291
Baseline multislice computed tomography measurements				
Aorta-RCA distance, mm	16.9 ± 3.8	17.3 ± 4.0	16.4 ± 3.4	0.115
Aorta-LMCA distance, mm	13.3 ± 3.7	13.1 ± 3.8	13.5 ± 3.6	0.476
Ascending aorta, mm	34.6 ± 4.1	34.2 ± 4.0	35.0 ± 4.2	0.386
Aortic annulus diameter, mm	24.0 ± 2.8	24.0 ± 2.8	24.1 ± 2.7	0.815
NCC-sinus of Valsalva diameter, mm	30.3 ± 5.6	30.3 ± 5.8	30.3 ± 5.4	0.998
RCC-sinus of Valsalva diameter, mm	28.3 ± 4.8	28.7 ± 4.9	27.9 ± 4.8	0.349
LCC-sinus of Valsalva diameter, mm	29.6 ± 6.8	30.0 ± 6.9	29.1 ± 6.7	0.394
Aortic annulus perimeter, mm	77.5 ± 8.2	77.4 ± 7.9	77.6 ± 8.5	0.892
Aortic annular area, mm^2^	455.9 ± 98.7	453.1 ± 96.4	459.2 ± 101.9	0.692

Data are expressed as mean ± standard deviation, frequencies (percentages), or as median (interquartile range) as appropriate

AR=aortic regurgitation; AV=aortic valve; IVSDD=interventricular septum diastolic diameter; LAD=left atrial diameter; LCC=left coronary cusp; LMCA=left mean coronary artery; LVEDD=left ventricular end-diastolic diameter; LVEF=left ventricular ejection fraction; LVESD=left ventricular end-systolic diameter; MR=mitral regurgitation; NCC=non-coronary cusp; RCA=right coronary artery; RCC=right coronary cusp; SPAP=systolic pulmonary artery pressure; TR=tricuspid regurgitation

### Clinical Results

Regarding the early clinical outcomes between the AA ≤ 48° and AA > 48° groups, the following results were obtained: requirement for PPMI (6.0% *vs.* 9.6%; *P*=0.283), new-onset stroke (4.0% *vs.* 2.6%; *P*=0.529), pericardial tamponade (2.7% *vs.* 1.7%; *P*=0.700), arrhythmias (16.8% *vs.* 18.3%; *P*=0.753), acute renal failure (4.7% *vs.* 5.2%; *P*=0.792), major bleeding (4.0% *vs.* 7.0%; *P*=0.292), major vascular complications (5.4% *vs.* 7.8%; *P*=0.420), coronary obstruction (only one case in the AA > 48° group; *P*=0.436), new-onset LBBB (31.5% *vs.* 34.8%; *P*=0.579), mild paravalvular leak (50% *vs.* 52.7%; *P*=0.692), moderate-severe paravalvular leak (2% *vs.* 4.3%; *P*=0.301), rehospitalization (22.8% *vs.* 25.2%; *P*=0.650), in-hospital mortality (4.7% *vs.* 8.7%; *P*=0.189), death at one-month follow-up (6.0% *vs.* 11.3%; *P*=0.125), and death at one-year follow-up (9.4% *vs.* 13.9%; *P*=0.252). None of these differences were statistically significant. Furthermore, among the 17 patients who experienced in-hospital mortality, all deaths were attributed to procedural and/or cardiac causes. Additionally, during the one-year follow-up, 23 patients succumbed to death as a result of cardiac causes. Other clinical results according to the AA grouping are summarized in Table 3.

**Table 3:** Procedural complications and clinical endpoints of the patients.

Complications		Aortic angulation			
		Overall	≤ 48°	> 48°	*P*-value
		n=264	n=149	n=115	
Technical success, n%		260 (98.2)	148 (99.3)	112 (98.2)	0.412
Permanent pacemaker, n%		20 (7.6)	9 (6.0)	11 (9.6)	0.283
New-onset stroke, n%		9 (3.4)	6 (4.0)	3 (2.6)	0.529
Pericardial tamponade, n%		5 (1.9)	2 (1.3)	3 (2.6)	0.656^*^
Arrhythmia, n%		46 (17.4)	25 (16.8)	21 (18.3)	0.753
Acute renal insufficiency, n%		13 (4.9)	7 (4.7)	6 (5.2)	0.847
Major bleedings, n%		14 (5.3)	6 (4.0)	8 (7.0)	0.292
Major vascular complications, n%		17 (6.4)	8 (5.4)	9 (7.8)	0.420
Coronary obstruction, n%		1 (0.4)	0	1 (0.9)	0.436^*^
New-onset LBBB, n%		87 (33.0)	47 (31.5)	40 (34.8)	0.579
Paravalvular leak, n%	Mild	114 (51.1)	65 (50)	49 (52.7)	0.692
	Moderate-severe	8 (3)	3 (2)	5 (4.3)	0.301^*^
Periprocedural MI, n%		2 (0.8)	1 (0.7)	1 (0.9)	N/A
Rehospitalization		63 (23.9)	34 (22.8)	29 (25.2)	0.650
Hospitalization day, IQR		3 (2-6)	3 (2-6)	3 (2-6.5)	0.856
In-hospital mortality, n%		17 (6.4)	7 (4.7)	10 (8.7)	0.189
Cumulative events#		45 (17.0)	21 (14.1)	24 (20.9)	0.147
First-month mortality, n%		22 (8.3)	9 (6.0)	13 (11.3)	0.125
First-year mortality, n%		30 (11.4)	14 (9.4)	16 (13.9)	0.252

Data are expressed as mean ± standard deviation, frequencies (percentages), or as median (IQR) as appropriate

IQR=interquartile range; LBBB=left bundle branch block; MI=myocardial infarction

#Cumulative events including permanent pacemaker, new-onset stroke, moderate-severe paravalvular leak, and in-hospital mortality

^*^Fisher’s exact test

A multivariable logistic regression analysis was conducted to identify predictors of cumulative events. The analysis included variables with a *P*-value < 0.2 from the univariable logistic regression analysis, such as AA, age, hypertension, chronic renal failure, and heart failure. In the multivariable logistic regression analysis, AA (odds ratio [OR]: 1.73, 95% confidence interval [CI]: 0.89-3.38, *P*=0.104), age (OR: 1.04, 95% CI: 0.99-1.10, *P*=0.099), hypertension (OR: 1.66, 95% CI: 0.82-3.33, *P*=0.155), chronic renal failure (OR: 1.82, 95% CI: 0.92-3.61, *P*=0.084), and heart failure (OR: 0.57, 95% CI: 0.27-1.21, *P*=0.145) were not found to have a significant association with cumulative events (Table 4).

**Table 4:** Independent predictors of in-hospital cumulative events# in univariable and multivariable logistic regression analysis model.

	Univariate analysis		Multivariate analysis	
	OR (95% CI)	*P*-value	OR (95% CI)	*P*-value
Aortic angulation	1.60 (0.84-3.06)	0.149	1.73 (0.89-3.38)	0.104
Age, years	1.05 (0.99-1.10)	0.069	1.04 (0.99-1.10)	0.099
Sex, female	0.77 (0.40-1.49)	0.453		
Hypertension	1.59 (0.81-3.12)	0.178	1.66 (0.82-3.33)	0.155
Diabetes mellitus	1.13 (0.54-2.36)	0.727		
CAD	0.83 (0.42-1.64)	0.603		
Atrial fibrillation	1.15 (0.54-2.44)	0.711		
Chronic renal failure	1.80 (0.92-3.52)	0.082	1.82 (0.92-3.61)	0.084
Heart failure	0.57 (0.27-1.19)	0.137	0.57 (0.27-1.21)	0.145

CAD=coronary artery disease; CI=confidence interval; OR=odds ratio

^#^Cumulative events including permanent pacemaker, new-onset stroke, moderate-severe paravalvular leak, and in-hospital mortality

## DISCUSSION

The present study aimed to investigate the impact of AA on periprocedural complications, in-hospital complications, and mortality of patients undergoing TAVR with transfemoral approach. The findings demonstrated that AA did not significantly influence clinical outcomes. Accurate imaging of the aortic annulus prior to TAVR is crucial for procedural planning^[13]^. Assessing the shape, calcification, diameter, and AA of the annulus helps in selecting the appropriate valve and reducing residual aortic regurgitation^[13]^. Therefore, preprocedural MSCT scanning, along with comprehensive case planning and implantation techniques, supports the use of SE valves in TAVR patients, aiming to minimize complications^[14]^. While echocardiography was previously employed for TAVR planning, MSCT has emerged as the preferred imaging modality for evaluating the AA and aortic anatomy^[15,16]^. Higher AAs, whether using BE or SE valves, require greater valve flexion, which may complicate accurate valve positioning and potentially increase the risk of post-implantation complications^[17,18]^. The presence of a horizontal aorta, representing extreme aortic root angulation, can pose significant challenges in correct bioprosthesis positioning during TAVR^[7]^. An angle > 70° between the plane of the aortic valve annulus and the horizontal plane/vertebrae is an exclusion criterion in clinical trials involving SE valves^[9]^. This is primarily due to the difficulties encountered with early generation valves during placement^[7]^. However, high AAs with new generation SE valves have not shown the same complications^[7]^. Another study conducted by Bob-Manuel et al.^[10]^ demonstrated that an increased AA had no significant impact on the shortor long-term outcomes of patients who underwent TAVR with new generation SE valves. Consistent with the literature, our study demonstrated that the AA did not significantly affect periprocedural complications and mortality during follow-up.

SE valves, especially in cases with high AAs, have been associated with challenges during valve crossing and coaxial insertion^[4]^. Device success was defined based on the VARC-3 criteria, which were updated in 2021. However, the definition of device success has varied across studies, leading to relatively variable results. A study^[19]^ examining the effect of AA on procedural success with SE Portico™ valves reported lower procedural success rates compared to previous studies. Nonetheless, our study showed that AA did not affect procedural success rates, which remained satisfactory for Evolut™ valves. The impact of AA on TAVR outcomes remains highly controversial and subject to debate. Sheriff et al.^[20]^ investigated the effect of aortic root angulation on post-TAVR outcomes in 50 patients who underwent SE CoreValve™ TAVR and found increased PVL rates with higher AAs. However, this study had limitations, such as a very small sample size and aortic evaluation performed using left ventriculography in a 30-degree right anterior oblique projection^[6]^. In recent years, MSCT has become more common for evaluating the AA, as in our study. The divergence between AA increase and PVL rates may be attributed to advancements in aortic evaluation and valve placement methods.

In the study conducted by Abramowitz et al.^[7]^, a retrospective analysis of 582 patients was performed to investigate the impact of AA on procedural success in early generation BE and SE valves. It was observed that high AA was associated with decreased procedural success rates in older generation SE valves, while the angle did not affect procedural success in BE valves^[7]^. High AA in SE valves was linked to increased rates of PVL, the need for post-dilatation or a second valve, and a higher incidence of valve embolization. Suboptimal valve positioning and the potential need for recapture and/or repositioning could potentially lead to more PVL, patient-prosthesis mismatch, and stroke^[7]^. These issues were believed to be related to more frequent stent deformation and asymmetrical placement caused by the long stent frame in older generation SE valves. However, these differences did not result in a significant increase in mortality or complications following SE TAVR. In contrast, in new generation SE valves, it was observed that AA did not affect procedural success and clinical outcomes, even at high angles^[7]^. Therefore, there is no harm in considering SE valves for patients with high AA, contrary to previous studies^[7]^. Similarly, in our study, no differences were found in terms of procedural success and short-term outcomes. Clinical outcomes, including device success, the need for a second valve or postdilatation, PVL rates, major complications, and mortality, were similar between the AA groups. These results can be attributed to the shorter stent frame and the flexibility of the delivery system in the relatively new generation valves. In extreme cases of high AA, the companion balloon technique can be utilized to facilitate the advancement of the unopened valve into the annulus, overcoming challenges in proper valve positioning^[21]^. For SE implantation in high AA cases, the use of a trap catheter in the delivery system and simultaneous advancement of both can be beneficial^[22]^. In our study, we employed new techniques and methods to prevent complications that may arise during valve advancement at high AAs. Additionally, the use of new generation repositionable SE valves may offer greater efficiency in cases with a high aortic opening. Our study included patients who underwent TAVR using completely new generation SE valves and underwent detailed imaging with MSCT prior to the procedure. Based on our findings, we concluded that AA does not significantly impact clinical outcomes in experienced centers, and it is not a determining factor for selecting SE valves.

In a retrospective analysis by Popma et al.^[5]^, involving 3,578 patients, the safety and efficacy of AA after SE TAVR were assessed. They reported that high angular grades were associated with poorer outcomes in older generation valves^[5]^. However, no association was found between AA and procedural success or clinical outcomes with the use of new generation SE valves. The authors attributed these findings to the utilization of the most up-to-date valve placement techniques^[5]^. The discrepancies observed between studies can be explained by the technological advancements in next generation devices. Over the past decade, improvements in the design of SE valves have facilitated their accurate positioning, even in patients with high AA^[23]^. Furthermore, advancements in TAVR implantation techniques, such as rapid ventricular pacing and the cusp overlap technique, have contributed to reduced rates of new patient-prosthesis mismatch in patients receiving new generation SE valves^[24,25]^. Consequently, new generation SE valves can be easily employed, even in cases with high AA.

### Limitations

Several limitations should be acknowledged in our study. Firstly, the study was conducted in a single center with a limited number of patients, and its retrospective nature introduces the possibility of selection bias. The inclusion of only transfemoral implantation cases and new generation valves further restricts the generalizability of the findings. Moreover, the calculation of AA was based on empirical angulation parameters recommended in commercial practice guidelines, and the use of more complex methods to assess thoracic and abdominal AA might yield more predictive results regarding procedural complications. Additionally, interoperator variability was not evaluated between the two groups, neglecting the potential impact of the operator’s experience on clinical outcomes.

## CONCLUSION

Our study demonstrates that increased AA does not significantly affect intraprocedural and periprocedural complications in patients receiving new generation SE valves. However, further evidence from multinational, multicenter, prospective, randomized studies is required to strengthen these findings.
